# Myocardial Infarction in an Ironman Triathlete: A Case for Advanced Lipid Testing in Cardiovascular Risk Assessment

**DOI:** 10.7759/cureus.103359

**Published:** 2026-02-10

**Authors:** Matt Mackler, Sabrina Brown, Sean Hu, Diego Alvarez, Petra Rocic

**Affiliations:** 1 Physiology and Pharmacology, Sam Houston State University College of Osteopathic Medicine, Conroe, USA; 2 Clinical Anatomy, Sam Houston State University College of Osteopathic Medicine, Conroe, USA; 3 Physiology and Pharmacology, University of Houston College of Medicine, Houston, USA; 4 Physiology, Sam Houston State University College of Osteopathic Medicine, Conroe, USA

**Keywords:** acute myocardial infarction, advanced lipid testing, ascvd risk, atherosclerosis, endurance athlete, lipid discordance, small dense ldl

## Abstract

Atherosclerotic cardiovascular disease (ASCVD) risk assessment relies heavily on low-density lipoprotein cholesterol (LDL-C), arterial blood pressure, and population-based risk calculators. Although effective for population screening, these approaches may underestimate risk in individuals with discordant lipid profiles when atherogenic particle burden is not captured by conventional testing. We report a 55-year-old male Ironman triathlete who suffered an acute myocardial infarction during competition. Evaluation showed posterior ST-segment changes, metabolic acidosis, transient hyperglycemia, and acute kidney injury. Coronary angiography revealed chronic total occlusion of the right coronary artery, complete occlusion of the left circumflex artery, and severe distal left anterior descending artery stenosis requiring multivessel percutaneous coronary intervention. Longitudinal outpatient testing demonstrated unremarkable risk factors, including mildly elevated total cholesterol and LDL-C, normal apolipoprotein B (apoB), and normal glycemic markers suggestive of low 10-year ASCVD risk. Advanced lipid testing after discharge showed markedly elevated LDL particle number (LDL-P) and increased small dense LDL (sdLDL), consistent with LDL pattern B. This case highlights how particle-based abnormalities may contribute to accelerated atherosclerosis despite reassuring conventional risk assessment and absence of guideline-defined lipid risk-enhancing factors.

## Introduction

Atherosclerotic cardiovascular disease (ASCVD) remains the leading cause of death worldwide, accounting for approximately one-third of all global deaths [1]. Preventive cardiology, therefore, relies on accurate risk stratification, most commonly using standard lipid panels and population-based risk calculators derived from pooled cohort equations.

Atherosclerosis begins when apolipoprotein B (apoB)-containing lipoproteins are retained within the arterial intima, where subsequent particle modification and vascular inflammation promote macrophage recruitment, foam cell formation, and plaque progression [2-5]. Low-density lipoprotein cholesterol (LDL-C) quantifies cholesterol mass contained within LDL particles but does not directly measure LDL particle number (LDL-P) or particle properties that influence arterial wall penetration and retention [6,7].

Current American College of Cardiology (ACC) and American Heart Association (AHA) cholesterol guidelines use LDL-C as the primary therapeutic target and incorporate selected lipid-related risk-enhancing factors, including markedly elevated apoB and lipoprotein(a), to refine risk estimation in borderline and intermediate-risk patients [8]. Routine evaluation typically includes a standard lipid panel, glycemic markers, and 10-year ASCVD risk estimation; however, LDL-P and LDL particle size are not routinely assessed and are not designated guideline-defined risk-enhancing factors [8].

A key limitation of cholesterol-centric assessment is LDL-C/LDL-P discordance. Discordance is frequently observed in obesity, metabolic syndrome, and diabetes, where insulin resistance and elevated triglycerides produce relatively cholesterol-depleted LDL particles such that LDL-C may underestimate atherogenic particle burden [6,7,9-11]. LDL-C/LDL-P discordance can also occur in individuals with otherwise favorable triglyceride and high-density lipoprotein (HDL) profiles, including some high-performing endurance athletes, in whom LDL-C may similarly underestimate long-term exposure to atherogenic apoB-containing particles [6,7,11,12].

Small dense LDL (sdLDL) particles are associated with increased atherogenic potential and are often reflected by an LDL pattern B phenotype. These sdLDL represent a recognized LDL subfraction associated with increased atherogenicity, partly related to reduced clearance and greater susceptibility to atherogenic modification. Compared with larger, more buoyant LDL particles, sdLDL is more likely to traverse the endothelium, demonstrate prolonged residence time, and undergo oxidative modification within the arterial wall processes central to plaque development [13-15]. These limitations may be particularly relevant in endurance athletes, who may have low calculated risk despite measurable coronary atherosclerosis in selected cohorts [12,16]. This report illustrates how elevated LDL-P and an LDL pattern B phenotype may contribute to severe multivessel coronary disease and acute myocardial infarction in an endurance athlete with reassuring conventional lipid testing and low estimated ASCVD risk.

This article was previously presented as a poster abstract at the American College of Physicians 2025 Texas Chapter Annual Scientific Meeting on November 9, 2025.

## Case presentation

A 55-year-old male Ironman triathlete collapsed during the cycling segment of a race on October 15, 2023, after acute chest tightness, dyspnea, and lightheadedness. He experienced cardiac arrest, became unresponsive, received bystander-initiated cardiopulmonary resuscitation, several rounds of defibrillation by EMS, and achieved return of spontaneous circulation prior to hospital arrival.

**Figure 1:**
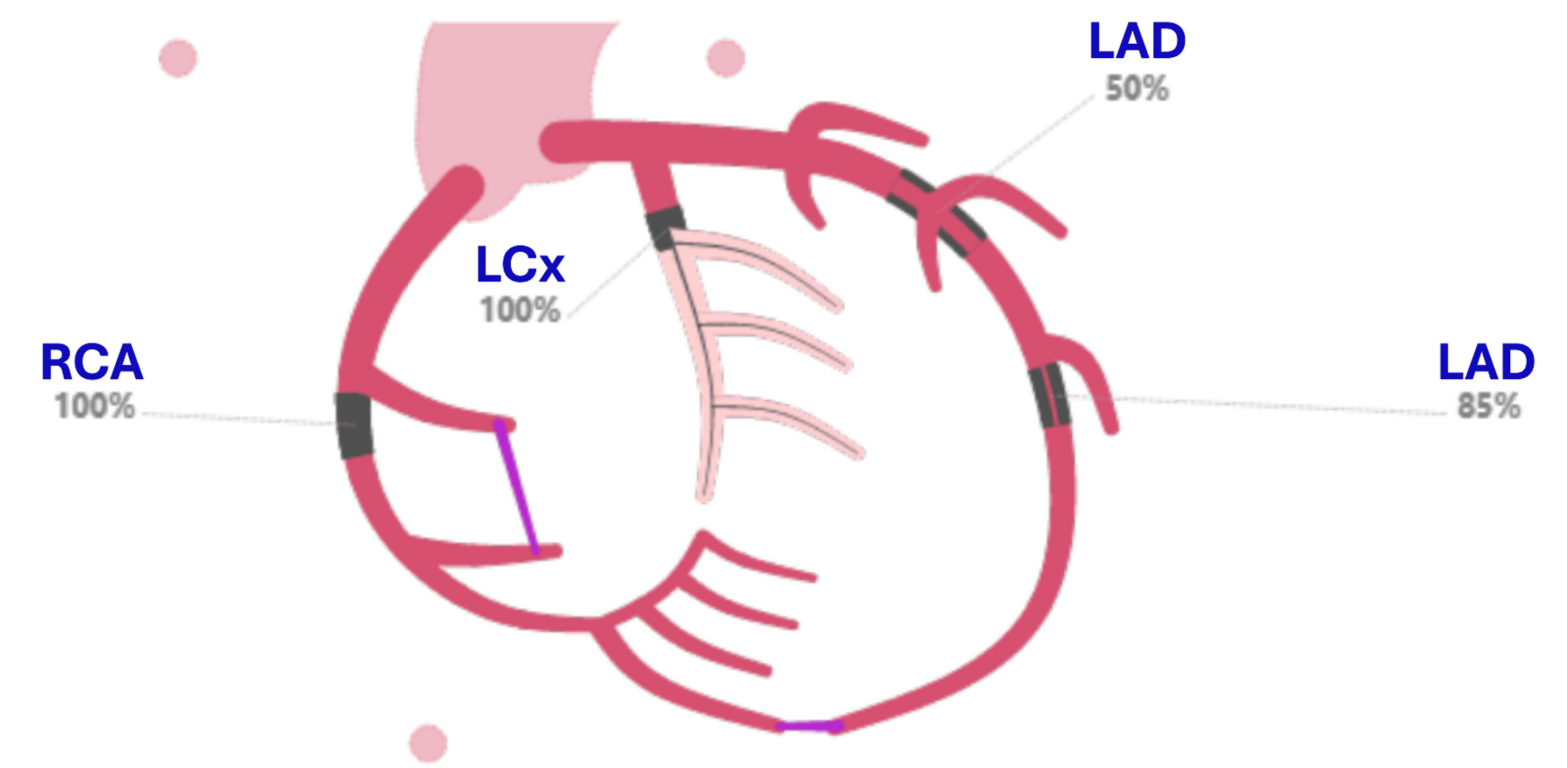
Coronary Angiographic Findings at Hospitalization Schematic depiction of coronary artery disease severity derived from the patient’s electronic health record and included for clinical illustration. The image demonstrates chronic total occlusion of the right coronary artery with collateral circulation, acute occlusion of the left circumflex artery, and severe proximal stenosis of the left anterior descending artery. These findings required multivessel percutaneous coronary intervention with placement of four coronary stents. The pink lines depict collateral vessels supplying the distal RCA beyond a chronic total occlusion, including bridging collaterals from the proximal RCA segment and intercoronary collaterals arising from the LAD artery. RCA: right coronary artery; LCx: left circumflex artery; LAD: left anterior descending artery; all images are de-identified and the original representative of the index procedure; source: patient electronic health record (used with consent)

Electrocardiography was consistent with a posterior ST-segment elevation myocardial infarction. Initial testing showed metabolic acidosis and transient hyperglycemia (serum glucose >240 mg/dL). Troponin rose from 0.08 ng/mL to a peak of 74.55 ng/mL. Coronary angiography demonstrated chronic total occlusion of the right coronary artery with collateralization, complete occlusion of the left circumflex artery, and approximately 85% distal stenosis of the left anterior descending artery. The patient underwent multivessel percutaneous coronary intervention with placement of four coronary drug-eluting stents (Figure 1).

Outpatient laboratory data from 2017-2023 showed total cholesterol 191-243 mg/dL, LDL-C 94-151 mg/dL, HDL-C 52-69 mg/dL, and triglycerides 81-139 mg/dL (Table 1). Advanced lipid testing was performed in the outpatient setting after recovery from the acute event. It revealed LDL-P 1518 nmol/L, increased sdLDL 308 nmol/L, reduced LDL peak particle size consistent with LDL pattern B, reduced large HDL particles, apolipoprotein B 84 mg/dL, and lipoprotein(a) in the normal-to-borderline range (Table 2).

**Table 1:** Longitudinal Standard Lipid Panel Assessments Longitudinal standard lipid panel values across serial measurements (2017–2023), demonstrating interval variability in calculated LDL-C and non–HDL-C, with post-event values shown for 2023. LDL-C: low-density lipoprotein cholesterol; HDL-C: high-density lipoprotein cholesterol; ACC: American College of Cardiology; AHA: American Heart Association

Marker	2017	2019	2020	2021	2023 (Post-Event)	ACC/AHA Reference Range
Total Cholesterol, mg/dL	191	220	243	219	167	Desirable <200; Borderline high 200–239; High ≥240
LDL-C, mg/dL	103	133	151	132	94	Optimal <100; Near-optimal 100–129; Borderline high 130–159; High 160–189; Very high ≥190
HDL-C, mg/dL	69	64	67	69	52	Low <40 (men), <50 (women)
Triglycerides, mg/dL	93	119	139	81	118	Normal <150; Borderline high 150–199; High 200–499; Very high ≥500
Non–HDL-C, mg/dL	122	156	176	150	115	Optimal <130; Borderline high 130–159; High 160–189; Very high ≥190

**Table 2:** Advanced Lipid Panel + Inflammation Testing (2023 Post-Myocardial Infarction) Advanced lipoprotein particle testing and inflammatory biomarkers obtained post-myocardial infarction (2023) demonstrated an elevated LDL particle number with a small, dense LDL pattern B phenotype. hs-CRP: high-sensitivity C-reactive protein; Lp-PLA2: lipoprotein-associated phospholipase A2; ApoB: apolipoprotein B; LDL-P: low-density lipoprotein particle number; LDL Phenotype Pattern B indicates predominance of small, dense LDL particles; LDL size Angstrom (Å) values in the low range are consistent with Pattern B; Lp(a): lipoprotein(a).

Marker	Result	Reference Range	Flag
hs-CRP	1.9 mg/L	<1.0 mg/L	High
Lp-PLA₂ Activity	110 nmol/min/mL	<124	—
Apolipoprotein B (ApoB)	84 mg/dL	<90 mg/dL	—
Lipoprotein(a) [Lp(a)]	65 nmol/L	<75 nmol/L	—
LDL Particle Number (LDL-P)	1518 nmol/L	<1138 nmol/L	High
Small LDL-P	308 nmol/L	<142 nmol/L	High
Medium LDL-P	344 nmol/L	<215 nmol/L	High
Large HDL-P	5967 nmol/L	>6729 nmol/L	Low
LDL Peak Size	216.2 Å	>222.9 Angstrom	Low
LDL Phenotype	Pattern B	Pattern A	Abnormal

Fasting glucose and hemoglobin A1c values were consistently within the normal range during this time period, with a transient increase in blood glucose, along with metabolic acidosis and evidence of acute kidney injury only in the immediate post-myocardial infarction period, consistent with stress physiology in the setting of acute myocardial infarction and cardiac arrest. The patient has no history of chronic kidney disease.

## Discussion

This case demonstrates that severe multivessel coronary artery disease and acute myocardial infarction can occur despite the absence of guideline-defined lipid or other risk-enhancing factors. Under current ACC/AHA guidelines, the patient’s calculated 10-year ASCVD risk remained below 5% across multiple assessments, placing him in a low-risk category; consequently, conventional evaluation likely underestimated his underlying atherosclerotic burden [8].

LDL particle phenotype classification (pattern A versus pattern B) is not incorporated into guideline-defined risk-enhancing factors. Pattern A is characterized by predominantly larger, more buoyant LDL particles, whereas pattern B reflects a predominance of smaller, denser LDL particles and is consistently associated with increased coronary artery disease and myocardial infarction risk [14,15,17]. Because sdLDL demonstrates adverse kinetic and compositional properties - including prolonged circulation time and greater susceptibility to oxidative modification - an LDL pattern B phenotype may confer higher ASCVD risk even when LDL-C appears only mildly elevated [13,15,17]. In this patient, advanced lipid testing demonstrated elevated sdLDL with an LDL pattern B phenotype, supporting a plausible biological explanation for extensive multivessel disease.

In addition to phenotype, LDL-C/LDL-P discordance represents a distinct mechanism by which conventional lipid assessment may underestimate risk. LDL-C quantifies cholesterol mass, whereas LDL-P reflects the concentration of circulating LDL particles, each containing a single apoB molecule, that are capable of arterial wall entry and retention [2,3,6]. When LDL particles are relatively cholesterol-depleted, LDL-C may appear modest despite substantially elevated LDL-P, resulting in greater cumulative exposure to circulating atherogenic particles than LDL-C alone suggests [6,7,9-12]. Consistent with this framework, the patient’s markedly elevated LDL-P implies a higher lifelong burden of circulating apoB-containing particles and an increased probability of arterial wall retention with progressive plaque accumulation over time [2,3,6,7].

LDL pattern B and LDL-C/LDL-P discordance may coexist and can reflect both genetic influences and triglyceride-rich lipoprotein remodeling, which promotes the formation of smaller, relatively cholesterol-depleted LDL particles. Under these conditions, LDL-C may appear only mildly elevated while LDL-P is disproportionately high, yielding discordance alongside a pro-atherogenic sdLDL-predominant phenotype [6,7,9-11,13].

Once retained within the intima, apoB-containing particles undergo modification and drive local inflammatory signaling, macrophage recruitment, and foam cell formation, accelerating plaque development and progression [2-5]. Beyond increased particle number, sdLDL may amplify risk through prolonged particle residence time, reduced clearance, and heightened susceptibility to oxidative modification, which may enhance macrophage uptake and promote more rapid plaque progression [13,15].

Although the lipid phenotype provides a biologically plausible substrate for long-term atherogenesis, the transient hyperglycemia observed during hospitalization was most consistent with stress hyperglycemia associated with acute myocardial infarction and cardiac arrest. Given repeatedly normal hemoglobin A1c values prior to the event, chronic dysglycemia was less likely to have contributed meaningfully to long-term plaque development [18].

This case also underscores challenges in cardiovascular risk assessment among endurance athletes. High levels of cardiorespiratory fitness, favorable lipid ratios, and absence of traditional metabolic risk factors commonly yield low estimated ASCVD risk, potentially contributing to under-recognition of progressive coronary disease until an acute event occurs. While endurance exercise confers substantial cardiovascular benefit for most individuals, observational data suggest coronary atherosclerosis can still be present in some lifelong endurance athletes even when conventional risk metrics appear favorable [12,16].

Taken together, these findings highlight that conventional lipid measures and guideline-based risk estimation may fail to identify biologically high-risk phenotypes in selected individuals. In this case, particle-based abnormalities provided a plausible explanation for severe multivessel coronary disease that was not anticipated by standard lipid testing or pooled-cohort risk estimates [6,7,9-12]. Long-term clinical follow-up was limited in this case and represents a potential limitation.

## Conclusions

This case illustrates that clinically significant atherosclerotic disease and acute myocardial infarction can occur despite mildly elevated LDL cholesterol, normal apolipoprotein B levels, and absence of guideline-defined lipid or other risk-enhancing factors. An LDL pattern B phenotype with sdLDL predominance and LDL-C/LDL-P discordance may represent biologically important risk pathways not captured by conventional lipid assessment. Although advanced lipid testing is not recommended for universal screening, it may be considered selectively in high-performing middle-aged endurance athletes. Advanced lipid testing in this population provides incremental risk information beyond conventional lipid measures and risk calculators.
